# Insights into the oral microbiota in human systemic cancers

**DOI:** 10.3389/fmicb.2024.1369834

**Published:** 2024-05-02

**Authors:** Lan Su, Rui Yang, Yanan Sheng, Saif Ullah, Yuheng Zhao, Hu Shunjiayi, Zhuo Zhao, Qingjing Wang

**Affiliations:** ^1^Key Laboratory of Artificial Organs and Computational Medicine in Zhejiang Province, Key Laboratory of Pollution Exposure and Health Intervention of Zhejiang Province, Shulan International Medical College, Zhejiang Shuren University, Hangzhou, Zhejiang, China; ^2^Department of Microbiology School of Biological Sciences, Quaid-i-Azam University, Islamabad, Pakistan; ^3^College of Biology and Environmental Engineering, Zhejiang Shuren University, Hangzhou, Zhejiang, China; ^4^School of Stomatology, Zhejiang Chinese Medical University, Hangzhou, China; ^5^Department of Computer Science and Engineering, University of Notre Dame, Notre Dame, IN, United States

**Keywords:** oral microbiota, systemic cancers, carcinogenesis, *Porphyromonas gingivalis*, *Fusobacterium nucleatum*

## Abstract

The oral cavity stands as one of the pivotal interfaces facilitating the intricate interaction between the human body and the external environment. The impact of diverse oral microorganisms on the emergence and progression of various systemic cancers, typified by oral cancer, has garnered increasing attention. The potential pathogenicity of oral bacteria, notably the anaerobic *Porphyromonas gingivalis* and *Fusobacterium nucleatum*, has been extensively studied and exhibits obvious correlation with different carcinoma types. Furthermore, oral fungi and viruses are closely linked to oropharyngeal carcinoma. Multiple potential mechanisms of oral microbiota-induced carcinogenesis have been investigated, including heightened inflammatory responses, suppression of the host immune system, influence on the tumor microenvironment, anti-apoptotic activity, and promotion of malignant transformation. The disturbance of microbial equilibrium and the migration of oral microbiota play a pivotal role in facilitating oncogenic functions. This review aims to comprehensively outline the pathogenic mechanisms by which oral microbiota participate in carcinogenesis. Additionally, this review delves into their potential applications in cancer prevention, screening, and treatment. It proves to be a valuable resource for researchers investigating the intricate connection between oral microbiota and systemic cancers.

## 1 Introduction

Currently, cancer is the most concerning disease in humans, with over 100 different known cancers affecting almost all regions of the body. Some well-studied periodontal microorganisms have become the focus of the association between oral microbiota dysbiosis and cancer development (Sun et al., 2020). The oral cavity constitutes one of the four major bacterial reservoirs in the human body, harboring a substantial abundance of microorganisms under normal physiological conditions. This configuration gives rise to a multifaceted microecological environment. In the physiological state of the human body, a profusion of microorganisms congregates, culminating in the creation of a complex microecological milieu (Xia et al., 2023). Oral microbiota comprises a consortium of microorganisms specifically adapted to inhabit the human mouth. The majority of these microorganisms engage in microbial physiological functions through coalescing on oral surfaces to create biofilms. With over 700 bacterial species colonizing the oral cavity, it constitutes one of the most intricate microbial communities within the human body.

These microbial communities inhabit a variety of niches, primarily including the mucous membranes, the rear of the tongue, dental structures, periodontal pockets, and the ever-changing realm of saliva. These oral microorganisms hold the potential to profoundly influence the initiation and progression of malignancies. This influence is enacted through mechanisms such as provoking chronic inflammation, generating carcinogenic metabolites, triggering epithelial-mesenchymal transition (EMT), modulating cellular apoptosis, and fomenting cellular proliferation.

Moreover, certain investigations have discerned that oral microorganisms can exert direct sway over host cell responses. Notably, *Clostridium* emerged with heightened transcriptional activity within oral squamous cell carcinoma (OSCC), surpassing other bacterial constituents. *Selenomonas* and *Prevotella* trailed closely, showcasing amplified activity within the OSCC context. Conversely, *Bacillus*, *Diplococcus* and *Neisseria* exhibited elevated activity within healthy sites. These findings intimate a potential correlation between oral microorganisms and cancer progression (Yost et al., 2018; Table 1). The purpose of this review is to explore the association between oral microbiota and systemic cancer, in order to have a positive impact on patient health in clinical practice.

**Table 1:** Oral microorganisms and pathogenic mechanisms of related diseases.

Microorganism	Diseases	Pathogenic pathways	Pathogenesis	References
*Pseudomonas aeruginosa*	OSCC	NF-κB pathway	Inducing inflammation through endotoxins and structural components; Reducing nitrite to nitric oxide; Secretion of LasI factor leads to downregulation of E-cadherin in tumor inhibition and carcinogenesis	Kakabadze et al. (2020)
*Porphyromonas gingivalis*	OSCC, Pancreatic Cancer, Esophageal Cancer	JAK 1/STAT 3 pathway	Stimulating the JAK 1/STAT 3 signaling pathway; Inhibition of ATP ligand binding to P2X7 receptor, Control the upregulation of mirNA-203; Adjusting Cell Cycle; Accelerate cell proliferation; Upregulate IL-8 and MMPs; EMT	Özlem et al. (2008), Pan et al. (2013), Ha et al. (2016), and Geng et al. (2017)
		TLR pathway, NF-κB pathway	Toll like receptors 2 and 4 bind to initiate the TLR signaling pathway, Activate NF-κB pathway, inducing tumor necrosis factor, IL-1α, IL-6, IL-8, etc. Forms an inflammatory microenvironment; With *Treponema denticola* and Fusetan bacteria, it produces peptide arginine deimidase PADase that degrades arginine, resulting in Point mutation of p53 and K-Ras	Rhodus et al. (2005), Olsen et al. (2016), and Wei et al. (2019)
		NF-κB pathway	Activate NF-κB pathway, inducing proliferation and metastasis of esophageal squamous cell carcinoma cells	Gao et al. (2016), Yuan et al. (2017), and Fan et al. (2019)
*Fusobacterium nucleatum*	OSCC, Colorectal	NF-κB, TLR4 pathway	Stimulating the expression of MMP-13 and MMP-9; Downregulation of p27 accelerates cell cycle; Increase the production of pro-inflammatory factors IL-6 and IL-8; Induced EMT	Uitto et al. (2005), Saito et al. (2012), Kauppila et al. (2013), Mima et al. (2015), Hsiao et al. (2018), and Geng et al. (2019)
			Invasion of intestinal Epithelium through Fusobacterium outer membrane protein A, adhesin A and Fusobacterium autotransporter 2, Activate NF-κ B and TLR4 signaling pathways; FadA adheres to E-cadherin in normal cells and cancerous epithelial cells, activating it β-Transcription pathways regulated by catenin	Sonnenburg et al. (2004), Roxana et al. (2013), and Yongzhi et al. (2017)
*Lactobacillus*	OSCC		Produce lactic acid and acidify the microenvironment together with other organic acids; Accumulation caused by changes in ROS and RNS activity	Piao et al. (2016), Hussain et al. (2003), and Karpinski (2019)
*Candida albicans*	OSCC	PI3K/Akt/NFκB, p38/JNK, ERK1/2/MAPK pathway	Produce Nitrosamine compounds; Produce hydrolysis related enzymes and virulence factors to invade tissues; Activating PI3K/Akt/NFκB p38/JNK, ERK1/2/MAPK signaling pathways	Sanjaya et al. (2011), Hafed et al. (2019), and Auperin (2020)
HPV	OSCC		HPV genes and gene products can interfere with cell cycle processes; HPV encodes E6 and E7 proteins, down regulates Histocompatibility gene expression, and affects innate immunity and adaptive immunity	Findik et al. (2019)
EBV	OSCC, Burkitt lymphoma, nasopharyngeal carcinoma		The mechanism of action in the carcinogenic process is still unclear	
*Helicobacter pylori*	Gastric Cancer, Pancreatic Cancer		Causing chronic gastritis and leading to gastric ulcers and atrophy, Persistent infection leads to cell damage of Gastric mucosa, cancer transformation; Deactivate ARID1A gene, resulting in cell Mitosis defect; Toxicity factors directly regulate the function of gastric epithelial cells and cause inflammation; Indirectly affect gastric epithelial cells, causing gastric ulcers	Ferreira et al. (2008), Chiba et al. (2010), Poplawski et al. (2013), and Inada et al. (2015)
			Lower acid production in patients and higher Nitrosamine level in individuals, leading to an increased risk of pancreatic cancer	
*Streptococcus mitis, Streptococcus granulosus*	Pancreatic Cancer		The decrease of abundance may cause Periodontal disease; *Streptococcus granulosus* may cause human inflammation and increase the risk of pancreatic cancer	Farrell and Gut (2012)
*Fusobacterium, Neisseria elongatum*	Pancreatic Cancer		A decrease in self abundance	
*G-bacteria*	Esophageal Cancer	NF-κB pathway	Participate in the innate immune process of the host and activate the nuclear transcription factor NF-κ B Classic Inflammatory Signal Pathway, Causing IL-1 β, IL-6, IL-8, TNF-α Increased expression; Activate inducible nitric oxide synthase and cyclooxygenase to reduce the peristaltic frequency of lower esophageal Sphincter	Abreu and Peek (2013)
*Streptococcus, Campylobacter*	Colorectal		Causing the occurrence of local microenvironment inflammation	

## 2 Enhancing oral microbiota’s role in OSCC carcinogenesis

### 2.1 The role of oral bacteria in advancing OSCC

**Figure 1:**
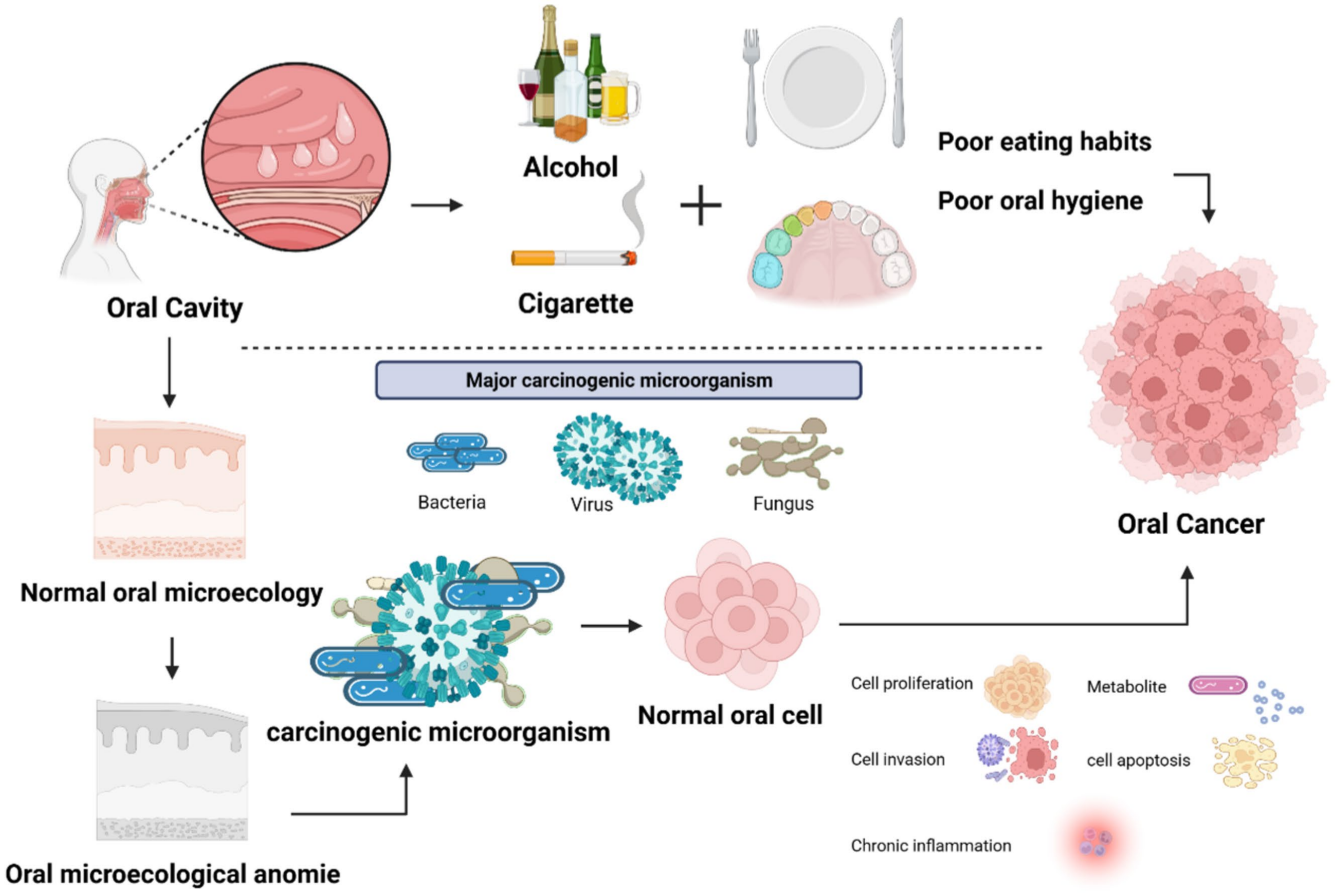
Factors influencing the occurrence and development of oral cancer. The factors that cause the occurrence and development of oral cancer: alcohol, cigarettes, poor oral hygiene or eating habits, oral microorganisms.

OSCC stands as the predominant malignancy within the head and neck domain, encapsulating over 90% of oral cancer cases. Despite therapeutic strides, patient survival remains dishearteningly low, and prognosis remains grim. For those diagnosed at advanced stages, the 5-year survival rate languishes below 50% (Manosha et al., 2016). The genesis of oral cancer is a nuanced interplay of multifarious factors. Conventional culprits like tobacco and alcohol endure as primary risk catalysts for OSCC. Equally implicated are compromised oral hygiene and dietary improprieties. Adding to this spectrum is the role played by the oral microbiota, contributing to a convergence of factors influencing the emergence of OSCC (Figure 1).

The association between oral microbiota and OSCC is robust. A slew of oral bacteria aligns closely with OSCC, the roster encompassing the likes of *Clostridium*, *Prevotella*, *Porphyromonas*, *Actinomyces, Clostridium, Haemophilus, Streptococcus,* and *Enterobacteriaceae* (Nagy et al., 1998). Within the oral Microorganism, several anaerobic strains are believed to be involved in the carcinogenic process (Sun et al., 2020). Among these, the prevalence of *Porphyromonas gingivalis* (*P. gingivalis*) and *Clostridium* proved to be significantly elevated in OSCC instances compared to their benign mucosal counterparts. Intriguingly, the survival rate of OSCC patients was correlated with the localization of *P. gingivalis* within tumor tissues (Nagy et al., 1998; Wen et al., 2020). Lactobacillus has a dual role in the development and advancement of OSCC. On one hand, it triggers apoptosis and enhances the expression of genes that suppress tumor growth. However, on the other hand, it also produces metabolites that have the potential to promote the formation and progression of cancer. This creates a complex interplay that influences the onset and advancement of OSCC (Karpinski, 2019). Furthermore, *Pseudomonas aeruginosa* is also involved in OSCC carcinogenesis. The bacterial endotoxins, such as Lipopolysaccharide (LPS), in conjunction with structural components like Flagella, play a role in shaping the organism’s inflammatory environment. They initiate inflammation by activating the NF-κB signaling pathway and also play a role in a complex chemical process. In this process, nitrite reductase transforms nitrite into nitric oxide, resembling a carefully choreographed chemical performance. The organism has the ability to secrete the LasI factor, which subsequently results in the suppression of a tumor-suppressing protein known as E-cadherin (Chattopadhyay et al., 2019; Cheng, 2020; Kakabadze et al., 2020).

### 2.2 Fungi and virus

Apart from bacteria, the oral terrain also harbors other microbial agents that can potentially drive OSCC, encompassing fungi, viruses and more. Notably, *Candida* emerges as a preeminent fungus in this milieu, a symbiotic opportunist that coexists with the host. *Candida albicans (C. albicans)*, a prevailing presence within the *Candida* genus, constitutes a common culprit behind infections. Intriguingly, recent investigations have unveiled the association between oral cancers and precancerous conditions, including white patches and oral lichen planus (Cosola et al., 2021). Initial researches have ascribed oral leukoplakia and oral lichen planus to Candida, intimating the genus’s partial culpability in these precancerous lesions (Werneck et al., 2015; Dilhari et al., 2016). *Candida* is capable of producing nitrosamine compounds. As potent carcinogens, nitrosamines stand out among the key chemical agents driving cancer, playing a pivotal role in the induction of OSCC (Sanjaya et al., 2011). A conspicuous correlation between the capacity of oral *candida* to metabolize alcohol into acetaldehyde and the fostering of cancer development corroborates the potential involvement of *C. albicans* in the carcinogenic tapestry (Alnuaimi et al., 2016). The main carcinogenic mechanism of OSCC caused by *Candida albicans* infection is through the Kirsten rat sarcoma virus oncogene homolog (KRAS) pathway and the E2 transcription factor (E2F) gene (Yang et al., 2023). In addition, mutations in tumor suppressor and DNA repair genes (such as p16, RAR-β2, TIMP3, BRCA1, and ERCC1) may have significant impact on the carcinogenic process due to *C. albicans* infection (Yang et al., 2023). Published studies have described the association between fungal communities and tumor transformation, and demonstrated a decrease in the diversity of individual fungal species that make up the oral fungal community in patients with oral squamous cell carcinoma. The increase in fungal species is related to the transformation of oral tumors, such as an increased incidence of the general Candida, Hannaela, and Giberella, which were detected in samples of OSCC tumor tumors (Vyhnalova et al., 2021).

Over the recent years, numerous studies have increasingly substantiated the influence of viruses in the progression of oral cancer (Yang et al., 2019; Rooper et al., 2020). Among these, human papillomavirus (HPV) stands out as a recognized carcinogenic factor (Findik et al., 2019) and is present in normal oral mucosa (Sankaranarayanan et al., 1997). Certain HPV types (16, 18, 31, 33, 35, and 39) have substantial carcinogenic potential, with their genes and products orchestrating interference in cell cycle processes. In addition, the twosome of oncoproteins encoded by HPV, notably E6 and E7, wield the ability to down-regulate the expression of histocompatibility genes. This action reverberates across both innate and adaptive immunity. 84% and 61.5% of patients diagnosed with OSCC tested positive for HPV and HPV-16, respectively. The presence of HPV increased the risk of developing OSCC by 2.82 times (Stasiewicz and Karpiński, 2022). Epstein–Barr virus (EBV) has been identified in oral and pharyngeal lymphoid tissues, with B lymphocytes serving as the primary reservoir for the virus. In addition to OSCC, EBV has been associated with oral diffuse large B-cell non-Hodgkin’s lymphoma, oral Burkitt’s lymphoma, and salivary gland epithelioma (Stasiewicz and Karpiński, 2022). A significant proportion (73.8%) of OSCC patient samples were positive for latent membrane protein-1 (LMP-1), a marker commonly associated with EBV-related malignancies, compared to only 19.1% in the control group. Furthermore, individuals infected with EBV have a 2.5 times higher risk of developing OSCC (95% CI = 1.23–5.36; Stasiewicz and Karpiński, 2022). Virome composition could be implicated in immune system development by regulating bacterial communities, thus indirectly contributing to the development of cancer (Tamayo-Trujillo et al., 2023).

## 3 Influence of oral microbes on the process of systemic cancers

### 3.1 Gastric cancer

Gastric cancer ranks as the fourth most prevalent malignancy globally and represents a prominent contributor to cancer-related mortality. Notably, samples obtained from individuals diagnosed with gastric cancer exhibited notably higher levels abundance of oral bacteria compared to those in benign stages (Coker et al., 2018). The oral cavity serves as a significant site for the colonization of *Helicobacter pylori (H. pylori)*. *Helicobacter pylori* can be detected in saliva, dental plaque, oral ulcers, oral tumors, etc. *Helicobacter pylori* infection plays an important role in the genesis of gastric cancer. The bacteria can alter the sensitivity of cells lining to stomach to a variety of exogenous dietary stimuli (Poplawski et al., 2013). Initially, it triggers chronic gastritis, progressing to gastric ulcers and atrophy. With persistent infection, it can inflict harm on the cells of the gastric mucosa, potentially culminating in their transformation into cancer (Poplawski et al., 2013). Simultaneously, *H. pylori* can deactivate the ARID1A gene, leading to abnormalities in cell mitosis, which fosters the advancement of gastric cancer (Inada et al., 2015). Moreover, *H. pylori* can directly modulate the function of gastric epithelial cells using its virulence factors (CagA, VacA). Additionally, it triggers inflammation and exerts indirect effects on gastric epithelial cells (Ferreira et al., 2008; Chiba et al., 2010).

### 3.2 Pancreatic cancer

Pancreatic cancer stands as one of the most fatal malignancies globally, contributing to approximately 8% to 10% of all digestive tract tumors. Within the group of individuals diagnosed with pancreatic cancer, the 5-year survival rate remains below 9% (Peters et al., 2017). Besides the recognized risk factors, oral microorganisms also play a role in the initiation and progression of pancreatic cancer. The main oral bacteria involved in carcinogenesis of pancreatic ductal adenocarcinoma are *P. gingivalis*, *Clostridium, Neisseria elongate (N. elongate)* and *Streptococcus mitis* (*S. mitis*; Riviere et al., 2010). Indeed, the occurrence of *F. nucleatum* within pancreatic tumors is associated with a poorer prognosis. Furthermore, *A. actino-mycetemcomitans, Alloprevotella, Leptotrichia,* and *Streptococcus* are also correlated with an elevated likelihood of developing PC (Stasiewicz and Karpiński, 2022). Additionally, *Granulicatella adiacens* was found to be elevated in PC cases (Stasiewicz and Karpiński, 2022). Under pathological circumstances, the levels of *N. elongate* and *S. mitis* decreased, while *Streptococcus* increased. *Streptococcus* possesses an inhibitory effect on periodontitis, and the reduction in its abundance could contribute to the onset of this condition. Additionally, it might trigger inflammation in humans, consequently elevating the likelihood of pancreatic cancer (Farrell and Gut, 2012). Individuals with elevated antibody levels of *P. gingivalis* face a more than twofold increased risk of developing pancreatic cancer compared to those with standard antibody levels (Dominique, 2013). *Porphyromonas gingivalis* initiates toll-like receptor (TLR) signaling pathway, activates NF-κB pathway, induces the expression of tumor necrosis factor, IL-1α, IL-6, IL-8 and other cytokines through binding with Toll-like receptors 2 and 4, thus forming an inflammatory microenvironment and promoting tumor occurrence and development (Rhodus et al., 2005; Olsen et al., 2016). Another mechanism of pancreatic cancer may be that the three red complexes of *P. gingivalis, treponema dentalis and Forsythian bacteria* produce peptidyl arginine deiminase PAD enzyme that degrades arginine, which causes p53 and K-Ras point mutations, leading to pancreatic cancer (Wei et al., 2019). *Helicobacter pylori* infection may also be a risk factor for pancreatic cancer (Yu et al., 2017). When comparing the serum levels of *H. pylori* IgG antibodies between patients with pancreatic cancer and healthy controls, it became evident that patients diagnosed with pancreatic cancer displayed elevated levels of *H. pylori* IgG antibodies (Kalaf et al., 2013; Chen et al., 2014). There exists a positive correlation between the occurrence of pancreatic cancer and the presence of gastric ulcers (Luo et al., 2007). *Helicobacter pylori* is capable of inducing gastric ulcers, resulting in decreased acid production and raised levels of specific nitrosamines. This interplay might consequently heighten the susceptibility to pancreatic cancer (Michaud, 2013).

### 3.3 Esophageal cancer

Esophageal cancer is listed as the eighth largest cancer in the world and the sixth leading predictor of mortality, with a 5-year survival rate of 15% to 25% (Ferlay et al., 2015; Gupta and Kumar, 2017). Elevated amounts of *P. gingivalis* were found in tissues afflicted with esophageal carcinoma. Moreover, esophageal squamous cell carcinoma tumor tissues exhibited even greater levels of *P. gingivalis* compared to adjacent paracarcinoma tissues and normal control sites. *In vitro* experiments, *P. gingivalis* triggered the NF-κB signaling pathway to induce the proliferation and metastasis of esophageal squamous cell carcinoma cells (Gao et al., 2016; Yuan et al., 2017; Fan et al., 2019). Esophageal cancer often coincides with an elevation in gram-negative bacteria. These bacteria produce substances like LPS and endotoxins, which might play a role in the innate immune response of the host. Among these components, LPS act as immunologically active agents. They have the capacity to initiate the conventional inflammatory signaling pathway through the nuclear transcription factor NF-κB. This, in turn, leads to an amplified production of inflammatory molecules such as IL-1β, IL-6, IL-8, and TNF-α. Additionally, it can activate inducible nitric oxide synthetase (iNOS) and cyclooxygenase enzyme (COX). This dual activation leads to a decrease in the peristalsis frequency of the lower esophageal sphincter, ultimately contributing to an elevated risk of esophageal cancer (Abreu and Peek, 2013).

### 3.4 Colorectal cancer

Colorectal cancer (CRC), which ranks among the top three contributors to cancer-related fatalities, has exhibited a rise in both occurrence and mortality rates over recent years. Recently, *F. nucleatum*, an anaerobic element found in both oral and intestinal symbiotic communities, has emerged as a noteworthy risk factor for CRC. Substantial variations in the oral levels of *F. nucleatum* and *C. difficile* were observed between individuals with normal conditions and those diagnosed with colorectal cancer. Notably, *F. nucleatum* facilitates the secretion of cytokines that have the potential to trigger the development of tumors (Yu et al., 2015). *Fusobacterium nucleatum* infiltrates the intestinal epithelial tissue using specific outer membrane proteins such as fusobacterium protein A (FomA), adhesion A (FadA), and fusobacterium self-transporter 2 (Fap2). This invasion triggers the activation of signaling pathways involving NF-κB and TLR4. Consequently, it spurs the proliferation of colon cancer cells, thus fostering the progression of tumors (Yongzhi et al., 2017). It possesses the ability to directly influence host cells by binding to E-cadherin in both normal and cancerous epithelial cells using FadA. This binding activates the β-catenin-regulated transcriptional pathway, leading to an upsurge in the expression of genes associated with cancer markers. Consequently, this process fosters the development of cancer (Sonnenburg et al., 2004; Roxana et al., 2013). Furthermore, it plays a role in facilitating the entry of non-invasive bacteria (like *Streptococcus* and *Campylobacter*) into cells. This leads to the development of localized microenvironmental inflammation, which in turn indirectly supports the progression of tumor cells and influences their prognosis (Tahara et al., 2014). *Fusobacterium nucleatum* promoted the development of cancer by enhancing the glucose metabolism of CRC cells, via activating the transcription of lncRNA ENO1-IT1 and increasing the binding efficiency of the transcription factor SP1 to its promoter region. The elevated ENO1-IT1 acted as a guide for KAT7 histone acetyltransferase, influencing the histone modification pattern on target genes such as ENO1, and ultimately impacting the biological function of CRC (Hong et al., 2021).

### 3.5 Lung cancer

Lung cancer is the cancer with the highest incidence rate and mortality in the world. Oral microbiome and cancer are closely related to lung and oral cavity, so oral microbiome and lung microbiome are highly related (Maddi et al., 2019). Lower levels of oral microbiome α Diversity may be associated with a higher risk of lung cancer and may serve as a prospective signal for lung cancer risk (Stasiewicz and Karpiński, 2022). *Fusobacterium nucleatum* may be a potential candidate microorganism for cancer biomarkers. *Porphyromonas gingivalis* may be involved in promoting the progression of malignant lung cancer (Stasiewicz and Karpiński, 2022). Periodontal disease may be mixed with smoking and positively correlated with the risk of lung cancer, but it is not an independent risk factor (Stasiewicz and Karpiński, 2022).

## 4 Carcinogenic mechanism

**Figure 2:**
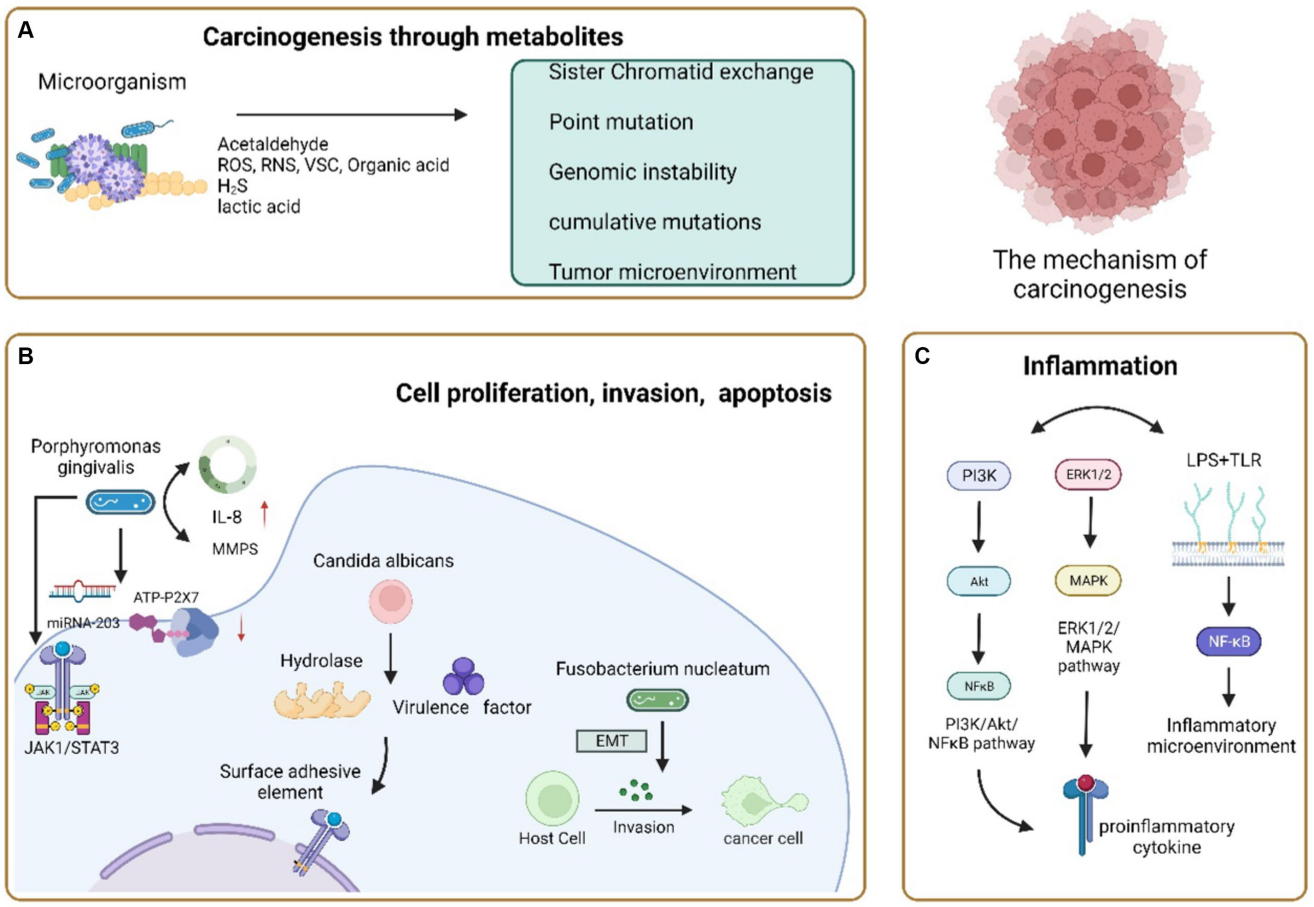
Carcinogenic mechanism of oral microorganisms. **(A)** Microorganisms cause cancer through their metabolites. Acetaldehyde, a common metabolite, can cause cancer occurrence and development through sisters Chromatid exchange, Point mutation, DNA adduct and excessive proliferation of epithelial cells; Reactive oxygen species, reactive nitrogen, volatile sulfur compounds, and organic acids alter their activity and accumulate carcinogenicity in inflammatory environments; H2S can cause genomic instability or accumulate mutations, activate proliferation, migration, and invasive signaling pathways, affect tumor growth and spread, and enhance tumor angiogenesis; Lactic acid can acidify the microenvironment together with other organic acids to form a Tumor microenvironment conducive to the growth of OSCC. **(B)** Carcinogenic pathways of microbial regulation of host cell proliferation, invasion, and apoptosis. *Porphyromonas gingivalis* induces cell apoptosis by over stimulating the JAK 1/STAT 3 signaling pathway, inhibiting ATP ligand binding to P2X7 receptors, and regulating miRNA-203 upregulation; Accelerate the proliferation of gingival epithelial cells by interfering with cell cycle regulation; Upregulation of IL-8 and Matrix metalloproteinase increases the invasiveness of Oral cancer cells. *Candida albicans* produces hydrolysis related enzymes and virulence factors to invade tissues, promote the proliferative epithelial response of oral mucosal cells, and make its dysplasia transform into cancer or induce apoptosis and necrosis of epithelial cells to cause cancer. *Fusobacterium nucleatum* can induce epithelial mesenchymal transformation and enhance the invasiveness of cells, achieving the goal of carcinogenesis. **(C)** Carcinogenic pathways of microorganisms through chronic inflammation. PI3K/Akt/NF-κB. The ERK1/2/MAPK signaling pathway is activated, and the secretion of pro-inflammatory cytokines promotes chronic inflammation and carcinogenesis; LPS binds to TLR-2 and TLR-4 on the host cell membrane, inducing the production of an inflammatory microenvironment to promote tumor growth.

In the context of cancer development, three key mechanisms are associated with oral microorganisms. Firstly, microorganisms can impact cancer pathogenesis by influencing processes like cell proliferation, cytoskeletal rearrangement, activating NF-κB, and preventing apoptosis. The second mechanism operates through the induction of chronic inflammation, which subsequently triggers the production of inflammatory mediators, playing a role in instigating or supporting various cancer-related events such as cell proliferation, genetic mutations, oncogene activation, and the promotion of angiogenesis. Lastly, a third mechanism involves certain microorganisms participating in cancer development by generating metabolites that have the potential to initiate cancer formation (Zhang et al., 2018; Figure 2).

### 4.1 Tumor microenvironment

Multiple studies have identified a correlation between the oral microbiota and the tumor microenvironment. The oral microbiota plays a significant role in various aspects of tumor dynamics, including initiation, progression, metastasis, and immune responses. This complex interaction within the tumor microenvironment (TME) is crucial for understanding cancer mechanisms. TME influences the development of tumors in multiple ways. For instance, it involves bacterial peptides found in both cancer and immune cells, where bacterial antigens imitate tumor antigens. Additionally, microorganisms play a role in prompting immunogenic cell death and activating signaling pathways through helper pattern recognition receptors. Furthermore, the metabolites produced by microorganisms also contribute to these processes, along with their impact on stimulation and inhibition of checkpoints (Gottesman, 2022). As an illustration, squamous cell carcinoma of the head and neck (HNSCC) is a prevalent condition that arises in areas like the oral cavity, oropharynx, larynx, and hypopharynx. These regions are composed of intricate tissues comprising not only tumor cells but also an encompassing matrix, that involves diverse types of mesenchymal cells and extracellular matrix (ECM; Hayes et al., 2018). Recent research has indicated that TME significantly impacts the growth and spread of squamous cell carcinoma of the head and neck (HNSCC; Joyce and Pollard, 2009). Genetic modifications within cancer cells of the TME, such as changes in TP53, NOTCH1, and distinct gene expression patterns, contribute to both the development of cancer and disruptions in the microenvironmental cells. These disruptions include heightened levels of reactive oxygen species (ROS), excessive cytokine production, and a transition between epithelial and interstitial states known as EMT (Boyle et al., 1993; Birner et al., 2000; Curry et al., 2014). In the progression of HNSCC, the adaptive immune responses are hindered through the excessive presence of cytokines. This excess leads to the apoptosis of T cells and alterations in how antigens are processed. The overexpression of crucial cytokines like transforming growth factor-β (TGF-β) plays a role in the development of processes such as EMTs, the emergence of immunosuppressive factors, and the formation of cancer-associated fibroblasts (CAFs; Cavallo et al., 2011; Curry et al., 2014). Within the TME, CAFs assume a paramount role in driving proliferation, invasion, and metastasis. Additionally, in the context of HNSCCs, tumor development is furthered by employing both glycolysis and oxidative metabolism (Curry et al., 2014). This intricate interplay underscores the importance of considering the oral microbiota’s role in shaping the tumor microenvironment and cancer progression. The tumor microenvironment can selectively promote the growth of specific bacteria, disrupting the balance between the resident oral microbiota and the host. This may be a crucial link through which commensal oral bacteria promote oral cancer. For instance, the genus *Peptostreptococcaceae incertae sedis*, which normally occupies only 0.04% of the normal oral buccal mucosa, increases to 0.36% in OSCC sites. Additionally, *Gemella morbillorum*, a facultative anaerobic *Gram-positive* coccus of the phylum Firmicutes, is highly associated with OSCC tumor sites (Zhang L. et al., 2020).

### 4.2 Oral microbial dysbiosis

Disruption of the balance in microbial flora leads to an imbalance known as oral microbial dysbiosis. This dysbiosis establishes a closely intertwined and mutually beneficial connection with the host. In the oral cavity, the regular colonization by microbial communities serves to prevent pathogenic microorganisms from attaching or impeding the growth of harmful bacteria. Additionally, these healthy microorganisms also play a role in countering and regulating the population of pathogens through antagonistic actions and quorum sensing mechanisms.

Simultaneously, the host’s immune system upholds a state of tolerance while curbing immune responses. This is achieved through the actions of mucosal dendritic cells and the release of anti-inflammatory cytokines like IL-10 and prostaglandin E2, the generation of T-regulatory cells also contributes to this process (Li et al., 2022). Consequently, the oral mucosal tissues achieve a harmonious and well-organized stability, balancing the survival needs of various microorganisms (Sami et al., 2020).

**Figure 3:**
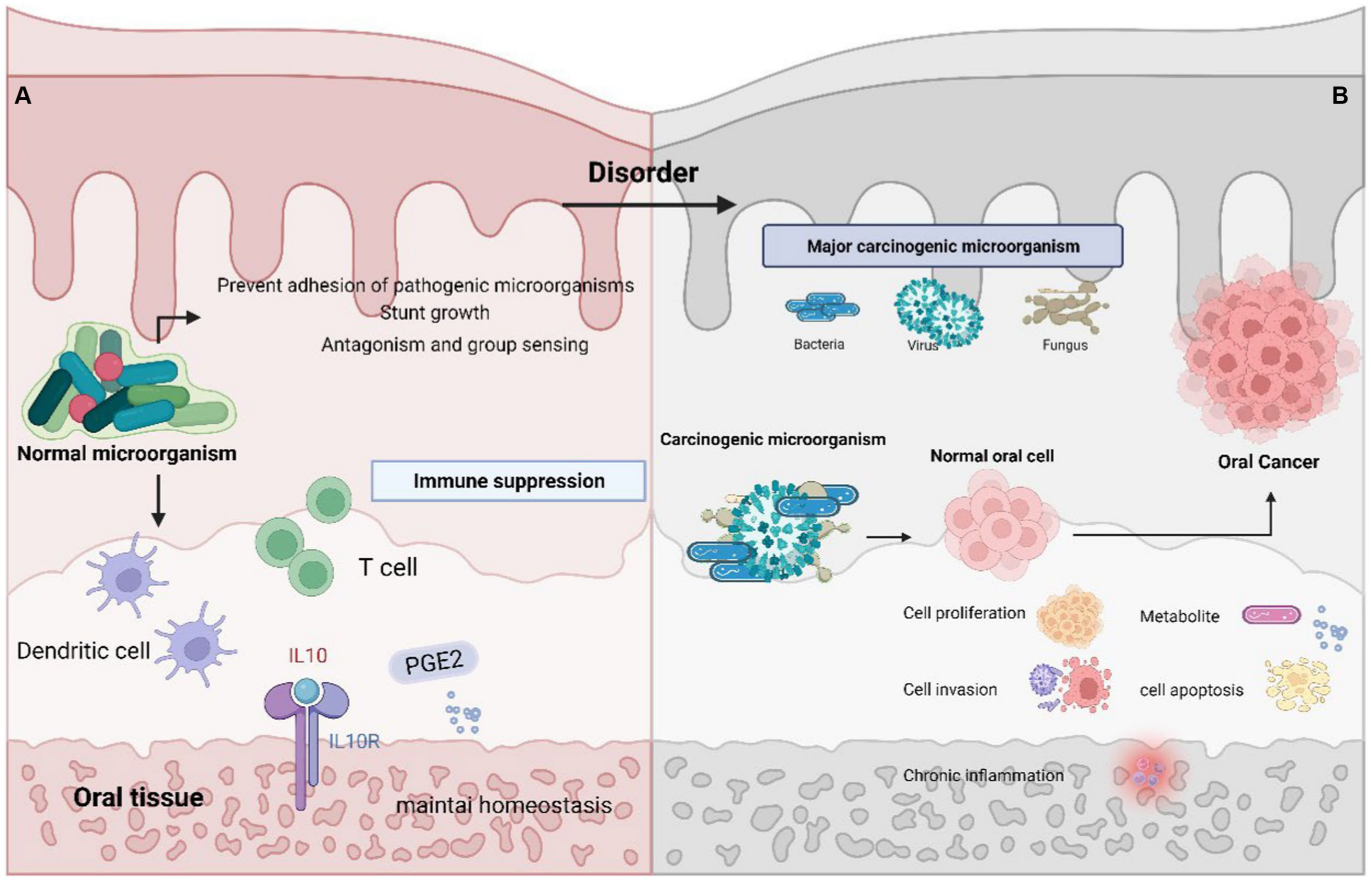
Oral microbiota balance and dysbiosis. **(A)** Normal oral environment can prevent pathogenic microorganisms from adhering to or hindering the growth of pathogenic bacteria, playing the role of antagonism and Quorum sensing; The host immune system maintains tolerance through the role of mucosal dendritic cells and the secretion of anti-inflammatory cytokines IL-10, Prostaglandin E2, T regulatory cells, etc. Maintain a stable oral microenvironment. **(B)** Abnormality of the oral microenvironment, where pathogenic bacteria begin to play a role and cause cancer through promoting cell proliferation, invasion, apoptosis, chronic inflammation, and the production of carcinogenic metabolites.

When the balance of microorganisms in the mouth is disrupted, it creates a favorable environment for harmful bacteria to become active and contribute to various oral diseases. For example, in conditions like tooth decay, these changes encourage the growth of bacteria that can produce and tolerate acidic conditions, such as *Streptococcus mutans*, *Lactobacillus*, and *Bifidobacterium* (Takahashi and Nyvad, 2011). Dysbiosis of oral microbiota may be involved in the development of oral cancer and other systemic diseases. The bacteria that undergo significant changes in oral cancer are mostly anaerobic, since hypoxia can lead to the recolonization of specific bacteria, which can promote the development of malignant tumors. When inflammation is uncontrolled or continuous, it can progress into a chronic state, which has the potential to promote the transformation of normal cells into cancerous cells (Zhang L. et al., 2020; Figure 3).

### 4.3 Regulation of host cell proliferation, invasion and apoptosis

Microorganisms can promote the progression of cancer by regulating apoptosis, activating cell proliferation, and enhancing cell invasion. *Porphyromonas gingivalis* employs multiple strategies to control apoptosis. For instance, it can prompt cell apoptosis by excessively activating the JAK1/STAT3 signaling pathway. This involvement in regulating mitochondrial apoptosis, cellular differentiation, migration, and proliferation is a notable aspect of *Porphyromonas gingivalis*’s impact (O'Shea et al., 2015)*. Porphyromonas gingivalis* can induce apoptosis of gingival epithelial cells by inhibiting the binding of ATP ligand to P2X7 receptor (Özlem et al., 2008). Furthermore, it influences cytokine signaling through the regulation of miRNA-203, which targets the 3’UTR region of SOCS3, leading to its reduced expression (Qin et al., 2012). As a result, this process triggers both apoptosis and the development of carcinogenesis.

Under long-term infection conditions, *P. gingivalis* can promote cell proliferation and change cell morphology by regulating cell cycle (Pan et al., 2013; Geng et al., 2017). Examination of the proteome has revealed that when gingival epithelial cells (GECs) are infected with *P. gingivalis*, alterations occur in the levels and phosphorylation of specific proteins. These changes disrupt the regulation of the cell cycle, fostering the shift from the S phase to the G1 phase. This is achieved by enhancing the presence of cell cycle cyclins (D and E) and consequently promoting a proliferative state in GECs (Kuboniwa et al., 2008; Pan et al., 2013). Moreover, *P. gingivalis* can enhance the aggressiveness of oral cancer cells through multiple mechanisms. This includes the elevation of IL-8 and matrix metalloproteinases (MMPs), as well as the induction of EMT (Ha et al., 2016). IL-8 can also contribute to cancer development by engaging in the elevation of zinc-dependent proteins. This, in turn, leads to the degradation of the extracellular matrix, facilitating the metastasis of malignant epithelial cells. Additionally, IL-8 plays a role in activating epidermal growth factor (EGF), which stimulates cell proliferation (Fitzsimonds et al., 2020). The existing pathology of the pancreas may induce the aggregation of oral microorganisms, and its pathological effects may cause pancreatic carcinogenesis (Andam and Hanage, 2015). Pathogens can enter the pancreas through various pathways, including blood flow, bile ducts, small intestine, and reflux into the pancreatic duct (Michaud et al., 2013). Therefore, it can be inferred that *P. gingivalis* originating from the oral cavity may spread to the cancer site or interfere with the immune system to accelerate the development of cancer (Sun et al., 2020).

*Candida albicans (C. albicans)* stands as the primary culprit behind oral candidiasis and has been linked to the emergence of OSCC. This microorganism generates enzymes for hydrolysis and virulence factors that aid in tissue invasion. Consequently, this invasive behavior triggers a heightened epithelial response in oral mucosal cells, which leads to their restrained growth and transformation into a cancerous state (Hafed et al., 2019). The primary mechanism involves *C. albicans* attaching to receptors on epithelial cells via surface adhesins like lectin-like sequence family and mycelium wall proteins. This attachment leads to adherence to oral epithelial cells. Following this initial interaction, *C. albicans* enters the upper cortex or submucosa of the host through two distinct mechanisms: endocytosis and active penetration. Subsequently, it triggers apoptosis and necrosis of epithelial cells, which ultimately contributes to the development of oral cancer (Marc et al., 2017; Peter, 2018). *Fusobacterium nucleatum* has the ability to either trigger epithelial-mesenchymal transition EMT or attach to proteolytic enzymes that originate from the host. This attachment subsequently boosts cellular invasion, facilitating the progression toward carcinogenesis (Renée et al., 2004; Nisticò et al., 2012). Moreover, *F. nucleatum* infection prompts the activation of protein kinase p38 within the infected cells, leading to escalated secretion of MMP-13 and MMP-9. This excessive secretion plays a role in tumor invasion (Uitto et al., 2005). Research has indicated that *F. nucleatum* can induce cell cycle arrest at the S phase by reducing the expression of p27. This, in turn, leads to heightened cell proliferation and involvement in the progression of cancer (Geng et al., 2019).

### 4.4 Chronic inflammation

The oral microbiota also exerts an impact on tumor development through its modulation of the host’s immune response. Prolonged infections stand out as contributors to cancer development, with approximately 16% of global cancer cases being directly linked to infections (Ojcius et al., 2016). The foremost hallmark of oral microbial infection is persistent inflammation, which assumes a pivotal role across all phases of cancer evolution. These stages encompass initiation, advancement, invasion, and metastasis. This is attributed to the prolonged inflammation resulting from ongoing environmental exposure (Uitto et al., 2005). Oral pathogens trigger inflammatory responses within the host, marked by heightened levels of cytokines, chemokines, and growth factors. These factors foster cell survival and growth, thus initiating the cancer development process (Geng et al., 2019). The cytokines generated during the inflammatory response significantly contribute to cancer formation by provoking mutations, destabilizing the genome, and affecting epigenetic processes. They further hinder apoptosis, promote angiogenesis, and disrupt normal functioning of inflammatory signaling pathways, ultimately leading to abnormalities (Zhang S. W. et al., 2020). Oral microorganisms like *P. intermedius* have the ability to generate harmful substances such as LPS, peptidoglycan, or lipoteichoic acid. These substances prompt the production of proinflammatory cytokines (Chattopadhyay et al., 2019). Additionally, cells secrete proteases that further encourage the production of these cytokines. Proteases can be activated through protease-activated receptors (PARs), serving as signaling molecules that degrade the host’s extracellular matrix. As a result, the physical immune barrier is compromised, the host’s immune response is disrupted, and conditions favorable for the emergence and progression of tumors are fostered (Zhang L. et al., 2020). *Fusobacterium nucleatum* has the capability to elevate the synthesis of pro-inflammatory cytokines, specifically IL-6 and IL-8. This, in turn, dampens the impact of immune cells, fostering an inflammatory environment that contributes to the development of oral cancer (Saito et al., 2012; Kauppila et al., 2013; Mima et al., 2015; Hsiao et al., 2018). *Streptococcus anginosus* also facilitates the advancement of oral cancer by generating pro-inflammatory cytokines like IL-1β and IL-6. Thus, it participates in the genesis of oral cancer by acting as a contributor to persistent inflammation in the oral region (Rai et al., 2021).

*Candida albicans* has the capability to infect and trigger the PI3K/Akt/NF-κB, p38/JNK, and ERK1/2/MAPK signaling pathways. This activation subsequently leads to the release of various antimicrobial peptides and pro-inflammatory cytokines, culminating in the establishment of a persistent state of inflammation (Auperin, 2020). In the context of invasive *C. albicans* infection, a small secreted protein called Sel1 can also initiate an inflammatory response dependent on TLR2/4. This, in turn, activates both the NF-κB and Mitogen-Activated Protein Kinase (MAPK) signaling pathways, resulting in the expression of proinflammatory cytokines and chemokines. Furthermore, *P. gingivalis* contributes to tumor development by modifying the nearby inflammatory microenvironment. The lipopolysaccharide (LPS) of *P. gingivalis* attaches to TLR-2 and TLR-4 receptors on the host cell membrane. This attachment triggers the activation of NF-κB through intracellular signal transduction pathways, ultimately leading to the production of IL-1α, IL-6, TNF-α, and IL-8. These cytokines promote the establishment of an inflammatory microenvironment that fosters the growth of tumors (Liu et al., 2020).

### 4.5 Metabolites

Acetaldehyde has been identified as a substance that can lead to various harmful effects on DNA, including sister chromatid exchange, point mutations, DNA damage, and the excessive growth of epithelial cells. It is classified as the first major category in human carcinogen by the International Agency for Research on Cancer (IARC; Brooks and Zahari, 2014; Mizumoto et al., 2017). Certain oral microbial species, such as *Streptococcus gordonii, Streptococcus sanguis, Streptococcus salivarius,* and *yeast*, play a role in this process. While these microorganisms are not inherently carcinogenic, they possess the enzyme alcohol dehydrogenase (ADH) which, in the presence of oxygen or low oxygen conditions, can produce acetaldehyde. This contributes to the potential harmful effects associated with acetaldehyde exposure (Feller et al., 2013).

Reactive oxygen species (ROS), reactive nitrogen species (RNS), volatile sulfur compounds (VSCs), and organic acids are examples of substances that can contribute to carcinogenic processes. Inflammatory responses can trigger the accumulation of ROS and RNS, primarily through alterations in the activities of NADPH oxidase and nitric oxide synthase (NOS). This response is influenced by molecules like TNF-α, IL-6, and TGF-β and its persistence can facilitate cancer progression (Hussain et al., 2003; Piao et al., 2016). Several oral microorganisms play a role in generating hydrogen peroxide (H2O2). These microorganisms include *Bifidobacterium juveniles, Lactobacillus acidophilus, Bifidobacterium fermentum, Bifidobacterium janssenensis, Bifidobacterium minutos, Streptococcus cogenii, Streptococcus oligomysaccharides, Streptococcus oral,* and *Streptococcus haematoides* (Abranches et al., 2018; Hampelska et al., 2020). Certain oral bacteria, including *P. gingivalis, P. intermedius, A. agglutinus,* and *F. nucleatum*, have the ability to generate volatile sulfur compounds (VSCs) such as hydrogen sulfide (H2S), methyl mercaptan (CH3SH), dimethyl sulfide [(CH3)2S], and dimethyl disulfide (CH3SSCH3). Among these, hydrogen sulfide (HS), a recognized genetic agent, holds the potential to induce genomic instability and cumulative mutations. Furthermore, it exerts influence on the growth and dissemination of tumors by activating signaling pathways that enhance proliferation, migration, and invasion. Notably, HS also promotes the development of new blood vessels that nourish tumors, a process known as tumor angiogenesis (Gaskins et al., 2007).

Studies have highlighted that a significant portion of the microbial groups found within OSCC tumor tissues belong to saccharifying and aciduric strains. These strains are characterized by their capacity to produce acidic compounds and their preference for acidic conditions (Hooper et al., 2006). The oral cavity harbors various lactic acid bacteria, including species like *Lactococcus*, *Bifidobacterium*, *Streptococcus, Colinococcus,* and *Amphicoccus*. These microorganisms contribute to the production of lactic acid, collectively fostering a tumor-friendly microenvironment for OSCC growth by collaborating with other organic acids to acidify the surroundings. This interplay significantly impacts the progression of OSCC (Karpinski, 2019). The production of organic acids not only leads to a reduction in pH levels but also carries implications for the immune response against tumors. Specifically, the lowered pH can impede the body’s ability to mount an effective anti-tumor immune response. Additionally, the generation of organic acids plays a role in stimulating the formation of blood vessels that supply tumors, contributing to tumor angiogenesis (Choi et al., 2013).

## 5 Potential of oral microbiota in cancer prevention, screening and therapeutic strategies

Oral microorganisms and their byproducts offer promise as potential indicators for the malignant transformation of cells, serving as candidates for screening various associated cancers. To date, over 100 potential biomarkers have been identified in saliva for the diagnosis of OSCC (Castagnola et al., 2011). Notably, the levels of certain microorganisms such as *Fusobacterium periodontium, Parvobacterium parvum, Streptococcus macrococcus, Haemophilus influenzae,* and *filamentous factor* display a gradual increase from stage I to stage IV of OSCC. This intriguing trend suggests that fluctuations in the abundance of these microorganisms might hold potential as markers for the process of oral carcinogenesis. Moreover, the involvement of oral microorganisms extends beyond diagnostics, as they also contribute to the prevention and treatment of various other types of cancers (Chen et al., 2020).

### 5.1 The role of oral microbiota in preventive screening

The Nrf2 activator sulforaphane (SFN) plays a pivotal role in the regulation of diverse biological functions encompassing cell cycle, growth, development, survival, cell death, and gene transcription. SFN is notably upregulated in many cancer cells and contributes to the process of carcinogenesis by influencing cellular activities such as proliferation, differentiation, and programmed cell death within tumors (Deng et al., 2011; Hu et al., 2019). Studies have pointed to a robust expression of SFN in tissues infected with *C. albicans*, particularly in OSCC tissues. This observation implies that SFN could potentially serve as a distinctive biomarker for predicting the malignant transformation triggered by *C. albicans* infection.

Oral leukoplakia, acknowledged as a precursor to oral cancer, often harbors the presence of specific microorganisms including *Bacteroidetes, Streptococcus,* and *Fusobacterium* (Hu et al., 2016; Hashimoto et al., 2019). Additionally, HPV has been detected positively in cases of oral leukoplakia. Collectively, this suggests that oral microorganisms like *Streptococcus, Fusobacterium*, and HPV might hold significance as indicators for monitoring oral precancerous lesions.

A potential method for early pancreatic cancer detection involves examining pathogens responsible for periodontitis, either through analyzing antibodies in the blood or assessing the oral microbiota. In a prospective study (Michaud et al., 2013), antibodies to oral bacteria in plasma samples were compared between individuals previously diagnosed with pancreatic cancer and healthy controls. The results indicated that individuals with elevated levels of antibodies to *P. gingivalis* faced double the risk of pancreatic cancer in comparison to those with lower levels (Fan et al., 2018). This points to the potential of oral microbiota as a predictive tool for pancreatic cancer. Furthermore, an intriguing finding was that *F. nucleatum* and its related genus, *Leptotrichia*, were associated with a reduced risk of pancreatic cancer. On the other hand, *N. elongate* and *S. mitis*, as constituents of the oral microbiome, could potentially contribute to the diagnosis of pancreatic cancer.

High recurrence rates are a significant concern in cancer cases (Serna et al., 2020), leading to the common use of postoperative adjuvant chemotherapy to mitigate the risk of recurrence. In colorectal cancer patients, the persistence of *F. nucleatum* infection after neoadjuvant chemoradiotherapy (nCRT) is linked to a high recurrence rate. As a result, *F. nucleatum* holds substantial promise as a biomarker to enhance the detection of colorectal cancer (Wong et al., 2016; Eklöf et al., 2017). Efforts have also been dedicated to identifying oral biomarkers associated with CRC detection, such as *Streptococcus* and *Prevotella* (Flemer et al., 2017). Understanding the bacterial species linked to cancer risk has the potential to significantly enhance early disease detection strategies.

### 5.2 Therapeutic strategies

A range of cancer treatment options are available, encompassing surgery, radiotherapy, nanomaterial therapy, and gene therapy. Interestingly, oral microorganisms have also been implicated in cancer treatment (Table 2). Certain bacteria from the *Lactobacillus genus* exhibit anti-cancer effects and can impede neoplastic transformation, by inducing apoptosis, boosting the expression of tumor suppressor genes, and modulating both adaptive and innate immune responses (Vyhnalova et al., 2021). One specific strain, *Lactobacillus rhamnosus*, holds the ability to counteract chronic inflammation linked to carcinogenesis. The combination of geniposide and *L. rhamnosus* has been shown to heighten apoptosis in HSC-3 cells. The consumption of nutrient-rich foods containing probiotic lactobacilli also contributes positively to tumor prevention. Probiotics mitigate the mutagenic effects of harmful agents while influencing the expression of proteins associated with cell proliferation, apoptosis, inflammation, and immune system activation (Kumar et al., 2010). Various experiments conducted on cancer cells have unveiled the anti-proliferative and pro-apoptotic properties of probiotics in these tumor models (Yu and Li, 2016). *Lactobacillus plantarum*, for instance, displays the ability to inhibit or activate the MAPKs and PTEN pathways, respectively (Asoudeh-Fard et al., 2017). This strain can be employed in cancer treatment by leveraging the induction of apoptosis in oral cancer cells through the application of *Lactobacillus plantarum* probiotic secretions. Cytotoxicity, denoting the toxicity that leads to cell death or destruction, is a crucial consideration. Certain virulent genes within bacteria hold potent lethality (Liu et al., 2016; Liang et al., 2017). For instance, streptolysin O serves as a cytotoxic agent capable of inducing antitumor activity in cell cultures, including 293 T cells (Doocey et al., 2022). The utilization of microbial toxicity for cancer therapy is currently an area of active investigation.

**Table 2:** Therapies of associated diseases caused by oral microbiota.

Microorganism	Diseases	Therapeutic mechanisms	References
*Lactobacillus rhamnosus*	OSCC	Inhibition of chronic inflammation associated with cancer transformation	
*Probiotics*	OSCC, Colorectal	Reduce the mutagenic effect of harmful substances; Regulate the expression of proteins related to cell proliferation, apoptosis, inflammation, and immune system activation	Kumar et al. (2010) and Yu and Li (2016)
		Reduce the activity of intestinal oncogenic enzymes related to the occurrence of colorectal cancer; Affects the maturation of immune cells and their products in the intestine; Reduce the incidence rate of diarrhea, enhance the integrity of intestinal barrier, and reduce inflammation	Rowland et al. (1998), Leblanc and Perdigon (2005), Ohkawara et al. (2007), Soltan et al. (2010) Liu et al. (2011), Nakanishi et al. (2013), Demers et al. (2014), and Zaharuddin et al. (2019)
*Lactobacillus plantarum*	OSCC	Inhibition and activation of MAPKs and PTEN pathways; Promoting apoptosis of Oral cancer cells by secretions	Asoudeh-Fard et al. (2017)
*Lactobacillus paracasei*	Pancreatic Cancer	Improving the efficacy of chemotherapy, increasing tolerance; Enhance IFN, transfer Th2 immune phenotype to Th1 immune phenotype, and enhance anti-tumor ability	Wörmann et al. (2014) and Chen et al. (2020)
*Lactobacillus*	Colorectal	Release bacteriocins to inhibit the growth of other bacteria; Inducing cytotoxicity and apoptosis, controlling the growth of antibiotic resistant strains and pathogenic bacteria, and maintaining intestinal homeostasis	

Bacteria can play a beneficial role in the chemotherapy of various cancers, including pancreatic cancer, colorectal cancer, and other diseases. For instance, in a mouse model of pancreatic cancer treated with gemcitabine and bevacizumab, the addition of *Salmonella typhimurium* improved the therapeutic effectiveness against pancreatic cancer (Hiroshima et al., 2014). *Lactobacillus paracasei*, a *Gram-positive* lactic acid bacterium found in the human gut, has shown promise as well. When added to a mouse model undergoing gemcitabine treatment, *L. paracasei* improved chemotherapy efficacy and increased tolerance (Chen et al., 2020). Further investigation has revealed that this enhancement is largely attributed to an increase in IFN levels, causing a shift from a Th2 immunophenotype to a Th1 immunophenotype, thereby boosting anti-tumor capabilities (Wörmann et al., 2014).

Bacterial therapy involves using genetically modified bacteria, weakened living bacteria, or substances derived from bacteria such as peptides (Stanley et al., 1994) with anticancer properties. Bacteriocins, peptides released by bacteria like *Lactobacillus*, exhibit antimicrobial characteristics that inhibit the growth of other bacteria (De Vos et al., 1997). Bacteriocins not only effectively suppress the growth of antibiotic-resistant and pathogenic bacteria, thereby maintaining intestinal equilibrium (Cotter et al., 2013; Dicks et al., 2018), they also can induce cytotoxicity and apoptosis, making them appealing candidates for cancer therapy. In animal models, probiotics have demonstrated the ability to significantly diminish the activity of enterogenic oncogenic enzymes implicated in colorectal carcinogenesis (Rowland et al., 1998; Leblanc and Perdigon, 2005; Nakanishi et al., 2013). Moreover, probiotics can impact immune cell maturation and their products within the gut, as well as curb tumor formation in systemic immune organs like lymph nodes and the spleen (Ohkawara et al., 2007; Soltan et al., 2010). These findings underline the potential of probiotics as dietary supplements with anti-tumor properties. Furthermore, probiotic administration has been shown to ameliorate adverse effects of chemotherapy and immunotherapy. In the context of colorectal cancer, positive effects encompass reduced incidence of diarrhea, enhanced gut barrier integrity, and diminished inflammation. As a result, probiotics could serve as effective adjuncts for both the prevention and treatment of colorectal cancer (Liu et al., 2011; Demers et al., 2014; Zaharuddin et al., 2019). The photosensitizer’s antibacterial and bactericidal characteristics have been effectively utilized in root canal treatment, periodontal treatment, and elimination of candidiasis, yielding positive outcomes (Prażmo et al., 2016).

## 6 Future prospects and conclusions

Oral microbiota play a multifaceted role in cancer development by influencing mechanisms like cell proliferation, invasion, immunosuppression, and inflammatory responses. Moreover, they contribute to cancer prevention strategies, screening methods, and anti-cancer treatments. Despite these advancements, certain challenges persist within current research. Firstly, there is a lack of comprehensive understanding and substantial direct evidence to support these underlying mechanisms. Additionally, while the association between oral microorganisms and cancer is apparent, definitive links have not been universally established. Therefore, further exploration into the precise carcinogenic mechanisms employed by these microorganisms is needed. The potential utilization of oral microorganisms as biomarkers for accurate cancer diagnosis and prevention remains an open question, along with the identification of microorganisms with precise therapeutic effects. Recent investigations highlight the significance of signaling pathways like JAK 1/STAT 3, PI3K/Akt/NFκB, p38/JNK, ERK1/2/MAPK, and the toll-like receptor family in the progression of oral cancer. These pathways suggest potential key avenues through which oral microorganisms can impact oral cancer. Additionally, exploring the potential of oral microbes as diagnostic and prognostic markers for cancer requires further investigation to unravel their full potential.

This paper delves into the assessment of how oral microbiota contribute to tumorigenic and carcinogenic mechanisms, as well as their potential as markers for detecting, screening, and preventing tumors. By thoroughly exploring the intricate connection between oral microbiomes and systemic human cancers, we can gain valuable insights that further our comprehension of cancer prevention and treatment. This deeper understanding has the potential to significantly impact clinical outcomes by reducing the mortality rates of affected patients.

## Author contributions

LS: Writing – review & editing. RY: Writing – original draft, Writing – review & editing. YS: Writing – original draft, Writing – review & editing. YZ: Writing – review & editing. HS: Writing – review & editing. ZZ: Writing – review & editing. QW: Writing – original draft, Writing – review & editing. SU: Writing – review & editing.
